# Epilepsy, neuroinflammation and cannabidiol What do we know thus far? 

**DOI:** 10.3389/fphar.2025.1749260

**Published:** 2026-01-05

**Authors:** Gabriela Pesántez Ríos, Emilio Perucca, Pasquale Striano, Roberto Caraballo, Ximena Pesántez Ríos, S. I. Pascual-Pascual, Galo Pesántez Cuesta

**Affiliations:** 1 Department of Pediatric Neurology, Centro Nacional de Epilepsia, Quito, Ecuador; 2 Department of Medicine (Austin Health), The University of Melbourne, Melbourne, VIC, Australia; 3 Department of Neuroscience, School of Translational Medicine, Monash University, Melbourne, VIC, Australia; 4 IRCCS Istituto Giannina Gaslini, Full Member of ERN EpiCARE, Genova, Italy; 5 Department of Neurosciences, Rehabilitation, Ophthalmology, Genetics, Maternal and Child Health, University of Genova, Genova, Italy; 6 Department of Neurology, Hospital de Pediatría Prof. Dr. Juan P. Garrahan, Buenos Aires, Argentina; 7 Department of Pediatric Neurology, Hospital Universitario La Paz, Madrid, Spain

**Keywords:** cannabidiol, drug-resistant epilepsy, epileptogenesis, Immunomodulation, neuroinflammation, seizures

## Abstract

Epilepsy is a common neurological disorder associated with recurring seizures that in about one-third of individuals are resistant to conventional medications. Neuroinflammation and alterations in the endocannabinoid system are involved in epileptogenesis and represent attractive targets for therapeutic interventions. Randomized placebo-controlled trials have shown that cannabidiol (CBD), one of the main active principles found in the *Cannabis* plant, significantly reduces seizure frequency in patients with Lennox-Gastaut syndrome, Dravet syndrome, and tuberous sclerosis complex (TSC). The FDA’s approval of a purified formulation of CBD (Epidiolex^®^) in 2018 marks a significant advance in the management of patients affected by these disorders. This review is focused on the activity of CBD as a neuroinflammatory modulator and antiseizure agent. Experimental evidence from *in vitro* and *in vivo* studies indicates that CBD reduces neuronal excitability and seizure activity by a wide range of mechanisms including, but not limited to, modulation of endocannabinoid, adenosine, GPR55, and TRPV1 receptors. It has also been shown that CBD’s molecular actions trigger immunomodulatory effects and inhibit neuroinflammation through reduced concentrations of proinflammatory cytokines, chemokines, reactive oxygen species (ROS) and neurotoxic factors in microglia. We discuss the evidence for CBD’s effects on neuroinflammation, and their implications for inhibition of epileptogenesis and seizure activity. We highlight how further elucidation of CBD’s mechanisms of action, and particularly its effects on neuroinflammation, could lead to a more rational, targeted utilization of this compound, guided by assessment of biomarkers predictive of clinical response. Improved understanding of CBD’s immunomodulatory and anti-inflammatory effects could also facilitate the design of controlled studies to confirm the potential value of this compound in the treatment of types of epilepsy beyond those for which regulatory approval has been already obtained.

## Introduction

Epilepsy is a chronic disease that affects approximately 65 million people, or about 1% of the world’s population (Reddy and Golub, 2016). Around 80% of individuals with epilepsy live in low- and middle-income countries (World Health Organization, 2019), resulting in significant socioeconomic burden for health services and society in general. Epilepsy has a major impact on the quality of life of individuals and their families, particularly when seizures are not fully controlled. In fact, most patients report having a poor quality of life due to the unpredictability of seizures, the risk of associated injuries, and the adverse effects of antiseizure medications (ASMs) (Moya et al., 2015; García-Galicia et al., 2014; García-Peñas, 2015; Luoni et al., 2011).

In the long term, the prognosis of epilepsy is mainly determined by the underlying etiology, which can be related to structural, genetic, infectious, metabolic, and immune causes (Scheffer et al., 2018). Early onset seizures are often associated with structural abnormalities visible on neuroimaging, which can have genetic (e.g., cortical developmental malformations, tuberous sclerosis) or acquired causes (e,g., ischemic injuries, trauma, infections) (Ochoa-Gómez et al., 2017; Ramos-Lizana, 2017). A study of 4,595 children found that more than 60% of epilepsies with seizure onset between 1 and 3 years of age had a structural cause, with the most prevalent etiologies being hypoxic-ischemic encephalopathy and tuberous sclerosis (Ochoa-Gómez et al., 2017; Pesantez et al., 2018). In adolescents and adults, focal epilepsies due to structural etiologies have also been correlated with a worse prognosis and greater probability of drug resistance, particularly in patients with comorbid conditions such as mental disability or psychiatric disorders (López González et al., 2015).

Despite the large number of available ASMs, about 30% of individuals with epilepsy do not achieve complete seizure control with pharmacological treatment (Kwan et al., 2010). This group of patients accounts for 80% of the cost of managing epilepsy (Begley et al., 2000). The International League Against Epilepsy (ILAE) defines drug resistant epilepsy as “as failure of adequate trials of two tolerated, appropriately chosen and used antiepileptic drug schedules (whether as monotherapies or in combination) to achieve sustained seizure freedom.” Sustained seizure freedom is defined as a period of 1 year without seizures or, for individuals with infrequent seizures, a period without seizures three times longer than the longest interictal interval prior to treatment (Kwan et al., 2010). Having drug resistant epilepsy does not imply intractability, because many of these patients may achieve seizure freedom with epilepsy surgery or with other treatment options such as neurostimulation or dietary therapies (López González et al., 2015). Moreover, some patients with drug-resistant epilepsy can achieve complete seizure control after trying additional ASMs, either as monotherapy or in combination. In these patients, the probability of achieving seizure freedom decreases progressively with increasing number of ASMs previously tried without success (Mula et al., 2019).

Cannabidiol (CBD) is among the latest ASMs introduced into the therapeutic armamentarium, and it has been found valuable in improving seizure control in individuals with pharmacoresistant epilepsy. The present article provides a concise overview of mechanisms leading to drug resistance in epilepsy, with a special focus on the role of neuroinflammation, and the influence of CBD on these mechanisms. Evidence of clinical benefit achieved with CBD in difficult-to-treat epilepsies will also be briefly reviewed, together with a discussion of future perspectives for a more rational and potentially broader use of this compound in the treatment of seizure disorders.

## Epileptogenesis and neuroinflammation

The mechanisms of drug resistance are multifactorial and are influenced by the patient’s age, the etiology of epilepsy, and previous treatments. More than 65% of patients with refractory epilepsy have an early onset of seizures (Moya et al., 2015), with a higher incidence in childhood, especially during the first year of life, where they often present complex clinical features, with infantile spasms and focal seizures predominating over generalized seizures (Ochoa-Gómez et al., 2017; Wilmshurst et al., 2015). Uncontrolled seizure activity can also lead to cognitive disorders (Pesantez et al., 2018; Cilio et al., 2014; Koo and Kang, 2017), particularly in patients in developmental and epileptic encephalopathies (DEEs) in whom a high burden of seizure activity can adversely affect neurodevelopment (Scheffer et al., 2018). To understand refractory epilepsy and its possible therapeutic targets, it is important to review the neurobiological mechanisms underlying the process of epileptogenesis.

**FIGURE 1:**
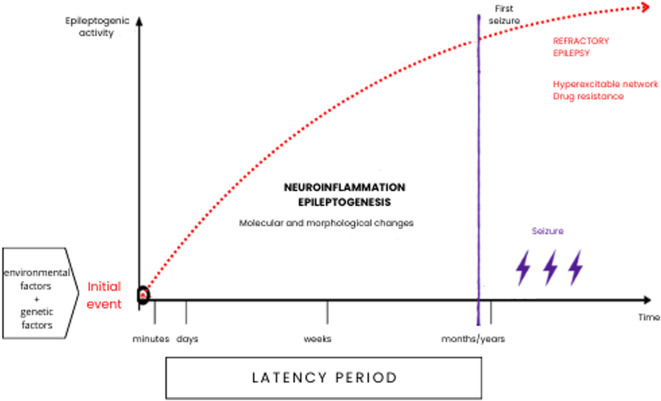
Epileptogenesis can occur throughout life and is a multifactorial process that, especially during infancy and early childhood. May have a genetic substrate, or it may be a secondary acquired process. Neuroinflammation and neuronal plasticity processes can cause morphological and molecular restructuring in neuronal circuits and play a fundamental role in the onset of seizure phenomena and drug resistance.

Long before the onset of the first seizure, there is a latent period, which can last for weeks, months or years during which neurobiological changes (epileptogenesis) gradually lead to the establishment of an epileptic condition (Figure 1). This period is characterized by a cascade of cellular and molecular events that can be triggered by various factors such as brain injury due to trauma, hypoxia, febrile seizures, infections, etc. Any of these factors, combined with a genetic predisposition, can trigger structural and/or functional changes that result in a hyperexcitable neuronal network capable of generating spontaneous epileptiform activity (Koepp et al., 2017; Rana and Musto, 2018), and subsequent onset of seizures and possible drug resistance (Suleymanova, 2021).

The process of epileptogenesis can be associated with a persistent inflammatory state in the neuronal microenvironment. This has been demonstrated in animal models of epilepsy where, after a critical insult, dendritic remodeling and synaptogenesis start to occur within 24 h. Microglial cells play a fundamental role in the initial proinflammatory response, releasing large amounts of cytokines detectable into the cerebrospinal fluid, such as interleukins IL-1β, IL-2, IL-6, and tumor necrosis factor α (TNF-α), among others (Rana and Musto, 2018; Vezzani et al., 2015). These, in turn, stimulate the proliferation of astrocytes and increase the expression of IL-1β, which indirectly promotes the release of glutamate, decreasing its reuptake in the synaptic cleft and generating greater hyperexcitability, consequently activating mechanisms of excitotoxicity, necrosis, and apoptotic activity (Koepp et al., 2017; Rana and Musto, 2018; Pardo et al., 2014; Choi and Koh, 2008). Recent evidence suggests that neuroinflammatory processes play a crucial role in drug resistance, as proinflammatory cytokines can modulate neurotransmitter receptors and upregulate efflux transporters at the blood-brain barrier, thereby reducing the efficacy of ASMs (Zabrodskaya et al., 2023; Vezzani et al., 2019).

Neuroinflammation is increasingly recognized as a shared mechanism that can contribute to the onset and persistence of seizures across multiple epilepsy syndromes. In animal models of infantile spasms and in several pediatric epileptic disorders, including Rasmussen encephalitis, infantile spasms, and FIRES, epileptogenic insults induce robust microglial activation, initiating sustained pro-inflammatory cascades and marked transcriptional changes. Beyond microglia, both neurons and astrocytes actively shape the inflammatory milieu through the release of cytokines, chemokines, and alarmins, thereby amplifying and perpetuating the neuroinflammatory response. These processes promote aberrant neurogenesis with mossy fiber sprouting, reactive gliosis, and alterations in the expression of GABAergic and glutamatergic receptors. Altogether, these neurobiological changes contribute to more severe and frequent seizures and are strongly associated with a higher level of refractoriness to antiseizure drug treatment (Pesantez et al., 2018; Pardo et al., 2014; Choi and Koh, 2008; Vezzani et al., 2019).

In temporal lobe epilepsy, increased levels of IL-1β and TNF-α have been reported, which could be associated with apoptotic cell death, excitotoxicity, necroptosis, and other regulated cell death pathways that contribute to the development of hippocampal sclerosis (Rana and Musto, 2018; Auvin et al., 2017; Vezzani et al., 2019). TNF-α increases the expression of AMPA receptors; promotes calcium uptake and amplifies glutamatergic transmission and number of glutamate receptors; it also induces endocytosis of GABA receptors, thereby reducing inhibition and promoting neurotoxicity and the maintenance of a hyperexcitable pathological network. This chronic hyperexcitability state also causes dynamic changes in the endocannabinoid system, mainly in the inhibitory homeostasis of CB1 receptors in the synaptic terminals of interneurons, causing prolonged disinhibition of the neural network (Rosenberg et al., 2015). In turn, IL-6 decreases long-term potentiation and neurogenesis in the hippocampus while increasing gliosis. All these conditions promote the maintenance of epilepsy and the onset of comorbidities. Sustained neuro inflammation has been linked to behavioral and cognitive comorbidities, including deficits in attention, memory, and emotional regulation, highlighting its systemic impact beyond seizure generation (Suleymanova, 2021; Rosenberg et al., 2017). Prolonged convulsive seizures, particularly convulsive status epilepticus, can also cause local biochemical and histological alterations around the primary seizure focus, which can lead to the appearance of new epileptogenic areas (secondary epileptogenesis). Altogether, these mechanistic insights provide a rationale for targeting glial signaling and endocannabinoid pathways early in the epileptogenic process.

The process of chronic neuroinflammation triggers cascades of immune responses, which may show clinical correlates. Evidence from human tissue supports this association: resected samples from focal structural epilepsies show marked glial activation and inflammatory signaling (Vezzani et al., 2015). Increased activation of IL-6 and MCP-1 (monocyte chemotactic protein-1) has been detected in patients with a history of epilepsy, compared to healthy controls. Additionally, increased pleocytosis without an active infectious process has also been reported in individuals who had a generalized tonic-clonic seizure, suggesting a causal relationship between neuroinflammation and both the onset and the progression of epilepsy (Choi and Koh, 2008; Auvin et al., 2017).

The latency period preceding the onset of seizures represents an important therapeutic window, with neuroinflammation mechanisms and neural network hyperexcitability being attractive targets to prevent the development and progression of epilepsy (Vezzani et al., 2019). In a prospective study, Kotulska et al. (2021) used electroencephalography (EEG) to detect epileptiform abnormalities in infants with TSC before the onset of clinical or electrographic seizures. They found that the probability of achieving seizure freedom was greater among those infants with epileptiform EEG abnormalities who received vigabatrin treatment prior to the onset of clinical or electrographic seizures compared with those who started treatment after a first seizure (Kotulska et al., 2021). Of note, imaging studies in animal models of epileptogenesis have identified evidence of brain inflammation before seizure appearance (Koepp et al., 2017). The efficacy of immunotherapy and adrenocorticotropic hormone (ACTH) in the treatment of infantile epileptic spasms syndrome could be due, at least in part to the anti-inflammatory and immunosuppressive properties of these compounds (Choi and Koh, 2008; Barker-Haliski et al., 2017).

## Is brain inflammation a relevant target for the efficacy of CBD in epilepsy?

Although herbal remedies based on components of the Cannabis plant have been used in the treatment of seizures disorders since ancient times (Perucca, 2025), it is only in the last decade that adequate evidence of clinical efficacy has been obtained from randomized double-blind placebo-controlled trials utilizing a pharmaceutical grade formulation of CBD (Epidiolex^®^) (Franco et al., 2021; Nabbout and Thiele, 2020). These trials showed that CBD is effective in improving seizure control when used as adjunctive therapy in patients with seizures associated with Dravet syndrome, Lennox-Gastaut syndrome and tuberous sclerosis complex (TSC). The results of these trials ultimately led to the approval of Epidiolex^®^ in the U.S, Europe and other countries for the treatment of seizures associated with these syndromes. Of note, many of the patients included in CBD trials were receiving concomitant treatment with clobazam. CBD inhibits the metabolic pathways of clobazam, leading to a 3.4 to 5-fold increase in the plasma concentration of the active metabolite N-desmethyl-clobazam (Nabbout and Thiele, 2020). While this interaction may contribute to the improved seizure control associated with CBD treatment and to some of its adverse effects, evidence has been if CBD does have an independent antiseizure effect (Bialer and Perucca, 2020).

**FIGURE 2:**
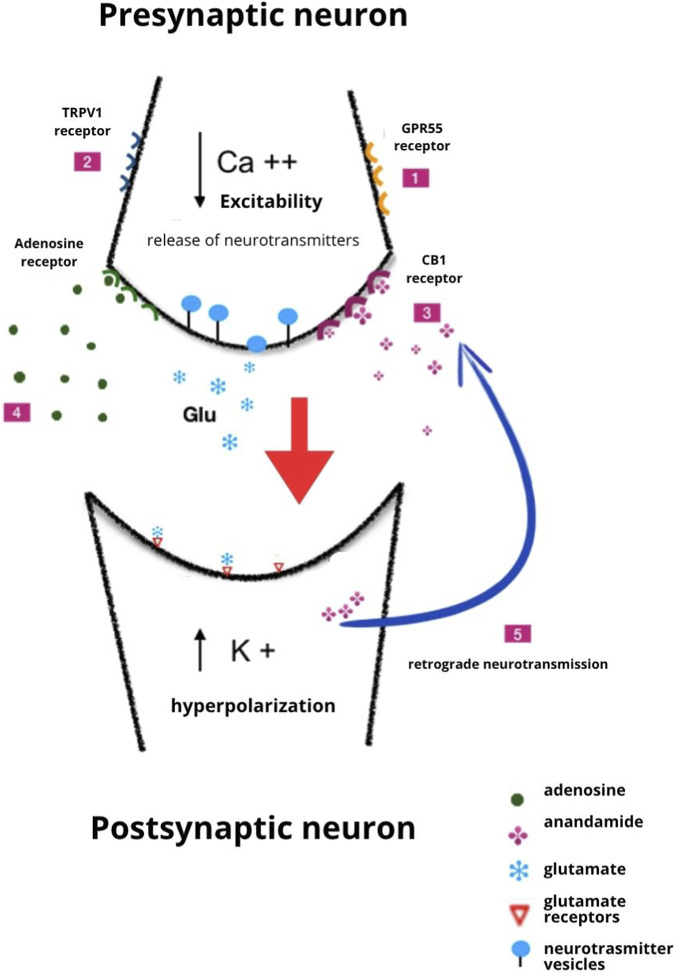
Illustration of several potential mechanisms by which CBD exerts antiseizure activity. 1. Antagonist action at the GPR55 receptor; 2. Desensitization of the TRPV1 receptor; 3. Inhibition of the metabolism of anandamide; 4. Inhibition of adenosine reuptake; 5. Induction of retrograde neurotransmission and long-term depression. The mechanisms shown here are not exhaustive, and other actions can contribute to the effects of CBD. Of note, some of the mechanisms shown in the figure probably contribute to CBD’s immunomodulating and anti-inflammatory effects. Modified from Gray and Whalley (2020), licensed under CC BY-SA.

The mechanisms by which CBD inhibits seizure activity are probably multifactorial and incompletely understood. Some of those considered to be particularly relevant are shown in Figure 2, but many others could play a significant role. The difficulty in identifying the primary actions responsible for CBD antiseizure effects is related to the complexity of CBD pharmacology. A 2019 review listed 18 different mechanisms of action reported for CBD, and the list has become even longer in the last few years (Swenson, 2025).

In addition to antiseizure activity, CBD possesses neuroprotective actions in a wide range of *in vitro* and *in vivo* experimental models. For example, in a rat model of transient global cerebral ischemia, CBD was effective in increasing brain-derived neurotrophic factor (BDNF) levels and in reducing ischemia-induced memory deficits, hippocampal CA1 neurodegeneration and deleterious changes in dendritic spine number and the length of dendritic arborization (Meyer et al., 2021). Similar results were obtained in a mouse model of brain ischemia induced by bilateral common carotid occlusion, where CBD reduced neuroinflammation and improved functional recovery (Mori et al., 2017). In a series of studies conducted in models of ischemic-hypoxic brain damage in newborn animals, CBD protected against tissue damage by modulating glutamate-induced excitotoxicity, oxidative stress, and inflammation (Pazos et al., 2012; Lafuente et al., 2016; Mohammed et al., 2017). In fact, modulation of inflammatory pathways in the central nervous system (CNS) is attracting considerable interest as one of the actions that could mediate CBD’s protecting effects against seizures (Cheung et al., 2019). This interest is justified not only by the studies discussed above linking neuroinflammation to epileptogenesis, epileptic activity and drug resistance, but also by evidence that dysfunction in endocannabinoid signaling can be implicated in epileptogenesis and brain inflammation (Cheung et al., 2019). Immunomodulating and anti-inflammatory effects of CBD in experimental models of injury to the brain or other organs have been demonstrated in many studies in addition to those mentioned above (Burstein, 2015; Yousaf et al., 2022). The protecting effects of CBD against brain inflammation take place at microglial level and lead to reduced concentrations of proinflammatory cytokines, chemokines, reactive oxygen species (ROS) and neurotoxic factors in microglia (Yousaf et al., 2022). The underlying mechanisms are not fully understood and may vary across experimental conditions and models. Mechanisms considered to be potentially relevant for prevention or suppression of neuroinflammation include modulation of CB2 receptors (Yousaf et al., 2022; Pazos et al., 2013), potentiation of adenosine signalling (Yousaf et al., 2022; Car et al., 2006; Mecha et al., 2013), scavenging of free radicals (Yousaf et al., 2022), inhibition of the NF-kappaB pathway and upregulation of the activation of the STAT3 transcription factor (Kozela et al., 2010; Dos-Santos-Pereira et al., 2020), modulation of the PI3K/mTOR signaling pathway (Lima et al., 2020), modulation of nitric oxide synthetase (NOS) expression and activity (Hooshm et al., 2025), modulation of TRPV1 ion channels (Socała et al., 2024) and others (Burstein, 2015; Yousaf et al., 2022; Hartmann et al., 2023).

Several studies have explored the relationship between CBD effects on neuroinflammation and protection against epileptogenesis and seizure activity. In an elegant study in the mouse model of epileptogenesis induced by bilateral intra-hippocampal injection of pilocarpine, Lima et al. (2020) found that CBD (30, 60 or 90 mg/kg, i. p., administered 30 or 60 min before pilocarpine) increased the latency and reduced the severity of pilocarpine-induced behavioral seizures, and prevented postictal changes, such as neurodegeneration, microgliosis and astrocytosis. In parallel experiments, some of which included use of transgenic mice, the authors also provided evidence that the antiseizure and neuroprotective effects of CBD were mediated by modulation of the PI3K/mTOR signalling pathway, possibly secondary to CB1 receptor antagonism. In another study, different modes and timing of administration of artisanal CBD in a mouse model of seizures induced by i. p. Injection of kainate were used to determine CBD effects on seizures, microglial activation and aberrant seizure-related neurogenesis (Victor et al., 2022). Prolonged treatment with CBD had no major effects on seizures in that study, but it reduced histologically documented neuroinflammation in the hippocampus as well as the number of ectopic neurons in the hippocampus after a seizure. Based on these findings, the authors considered CBD as a potentially useful adjunctive treatment in interventions aimed at preventing epileptogenesis. Somewhat similar results have been reported recently in a genetic mouse model of CL2 disease (Dearborn et al., 2022). Animals treated with CBD orally for 6 months (100 mg/kg/day) showed decreased markers of astrocytosis and microgliosis, but no effects were seen on seizure frequency or neuron survival. Whether higher doses of CBD might have been effective to also reduce seizures and neurodegeneration is unclear. Overall, while evidence from animal studies does confirm an antagonistic action of CBD on neuroinflammation in seizure models, the role of anti-inflammatory and immunomodulating mechanisms in mediating CDB’s antiseizure activity requires further evaluation.

Of note, evidence is now starting to emerge that CBD could be clinically beneficial in epileptic conditions where immune and inflammatory mechanisms are known to have a prominent role. Preliminary observations suggest that CBD can be valuable in the management of patients with refractory status epilepticus (Duda and Reinert, 2022), febrile infection-related epilepsy syndrome (FIRES) (Fetta et al., 2023), and Rasmussen syndrome (Prager et al., 2022). Additionally, a recent study in 8 adults with treatment resistant epilepsy (6 with focal epilepsy, one with idiopathic generalized epilepsy, and one with epilepsy undetermined whether focal or generalized) provided indirect evidence that CBD can reduce brain inflammation (Sharma and Szaflarski, 2024). Magnetic resonance spectroscopic imaging and thermometry (MRSI-t) was used as a biomarker of brain inflammation in these patients, based on the hypothesis that brain temperature elevation can serve as a surrogate marker for the biological processes associated with neuroinflammation. The study showed that patients had abnormally elevated peak temperatures within their seizure onset zone, and that these temperatures decreased significantly after 12 weeks of treatment with CBD (starting dose 5 mg/kg/day, maintenance dose not reported). CBD treatment was also associated with reduced seizure severity scores. While these results are consistent with the hypothesis of CBD reduces brain inflammation in patients with uncontrolled seizures, they should be interpreted cautiously because of many study limitations, including a small sample size, inclusion of patients with different types of epilepsy, lack of a randomized control group of patients with epilepsy not treated with CBD, and uncertainty about the validity of MRSI-t as a marker of neuroinflammation. Yet, the study highlights the potential value of assessing clinical biomarkers of brain inflammation, and their application in prospective clinical investigations on the mode of action of CBD.

## Conclusions and future perspectives

The last decade has seen impressive advances in understanding the value of CBD in the management of epilepsy. High quality investigations in experimental models have demonstrated its antiseizure activity irrespective of use of concomitant medications (Devinsky et al., 2014; Friedman and Devinsky, 2015; Devinsky et al., 2024). Its clinical efficacy in the treatment of seizures associated with Dravet syndrome, Lennox-Gastaut syndrome and TSC has been established by randomized placebo-controlled trials, leading for the first time to international regulatory approval of pharmaceutical grade CBD (Franco et al., 2021; Nabbout and Thiele, 2020). Increasing evidence is also accumulating that CBD efficacy may extend to a wide range of other epileptic conditions, ranging from DEEs to other rare syndromes, as well as common focal and generalized epilepsies in children and adults (Pesántez Ríos et al., 2022; Pesántez Ríos et al., 2017; Kochen et al., 2023; Chemaly et al., 2025; Cerulli Irelli et al., 2025; Perulli et al., 2025; Hollander et al., 2025; Saranti et al., 2025; Reyes et al., 2025). Despite these advances, important unmet needs and questions remain to be addressed. Treatment with CBD remains based on a trial-and-error approach, and we do not have reliable measures to predict patients who will benefit from treatment. We also need more data on (and predictors of) the effect of CBD on epilepsy co-morbidities, which are particularly common in individuals with DEEs (Vasquez and Fine, 2025). We also need rigorous, well controlled studies to confirm the efficacy of CBD in a broader range of epileptic syndromes and disorders.

Further research on the mechanisms of action of CBD could provide valuable clues to address many of the unmet needs mentioned above. It would be important to determine to what extent CBD’s immunomodulating and anti-inflammatory actions in the CNS contribute to its therapeutic effects, and whether such contribution differs in relation to age, epilepsy syndrome, epilepsy etiology and other individual factors. Coupled with the development of biochemical, electrographic, and imaging biomarkers to reliably identify the presence of neuroinflammation (Aguilar-Castillo et al., 2024; Shoukair et al., 2025), this information could allow to predict which patients are more likely to benefit from CBD treatment. It could also be valuable in determining additional indications to be pursued in future regulatory trials, and in selecting patients to be enrolled in such trials. Further research is also indicated to determine CBD’s potential in preventing epileptogenesis through inhibition of neuroinflammation. The availability of *in vivo* biomarkers of neuroinflammation could be very useful in these studies and might guide patient selection should a clinical trial of epilepsy prevention (or disease modification) be designed.

Overall, there are many attractive perspectives for a more rational, targeted use of CBD in the future, both for approved indications and for off-label conditions where evidence-based use could be justified. One problem, particularly in Latin America and other resource-restricted settings, is that the availability of pharmaceutical grade CBD is limited, and its cost is prohibitive for some sectors of the population. Use of cheaper formulations of artisanal medical CBD could be valuable in these settings (Tzadok et al., 2022; Pietrafusa et al., 2019). Due to the increased interest of caregivers in the use of *Cannabis*-derived products for the treatment of epilepsy, a pilot study was conducted in Ecuador since 2015 to evaluate the value of low doses of pure CBD added to pre-existing ASMs in the treatment of patients with severe epilepsies. After at least 12 months of treatment, the study reported significant reduction in seizure frequency, duration and intensity, and many patients showed neurocognitive improvements (Pesántez Ríos et al., 2022; Pesántez Ríos et al., 2017). While these data are encouraging, when using non-pharmaceutical grade CBD, it is important to be aware that the quality of products in the market, many of which can be purchased online, can be extremely variable. Studies conducted in the U.S. and other countries have clearly shown for some products the actual CBD content may differ wildly from that stated in the label, and some products may even be contaminated by pollutants (Pavlovic et al., 2018; Gurley et al., 2020; U.S. Food and Drug Administration, 2025). If non-pharmaceutical grade of CBD is not accessible or affordable, physicians and caregivers should ensure that the alternative product used is obtained from verifiable sources and meets adequate quality standards in terms of CBD content, stability and purity.
